# Secondary Use of Clinical Data in Data-Gathering, Non-Interventional Research or Learning Activities: Definition, Types, and a Framework for Risk Assessment

**DOI:** 10.2196/26631

**Published:** 2021-06-08

**Authors:** Martin Jungkunz, Anja Köngeter, Katja Mehlis, Eva C Winkler, Christoph Schickhardt

**Affiliations:** 1 Section for Translational Medical Ethics, Department of Medical Oncology National Center for Tumor Diseases Heidelberg University Hospital Heidelberg Germany; 2 Section for Translational Medical Ethics National Center for Tumor Diseases German Cancer Research Center (DKFZ) Heidelberg Germany

**Keywords:** secondary use, risk assessment, clinical data, ethics, risk factors, risks, privacy, electronic health records, research, patient data

## Abstract

**Background:**

The *secondary use of clinical data in data-gathering, non-interventional research or learning activities* (*SeConts*) has great potential for scientific progress and health care improvement. At the same time, it poses relevant risks for the privacy and informational self-determination of patients whose data are used.

**Objective:**

Since the current literature lacks a tailored framework for risk assessment in *SeConts* as well as a clarification of the concept and practical scope of *SeConts*, we aim to fill this gap.

**Methods:**

In this study, we analyze each element of the concept of *SeConts* to provide a synthetic definition, investigate the practical relevance and scope of *SeConts* through a literature review, and operationalize the widespread definition of risk (as a harmful event of a certain magnitude that occurs with a certain probability) to conduct a tailored analysis of privacy risk factors typically implied in *SeConts*.

**Results:**

We offer a conceptual clarification and definition of *SeConts* and provide a list of types of research and learning activities that can be subsumed under the definition of *SeConts*. We also offer a proposal for the classification of *SeConts* types into the categories *non-interventional (observational) clinical research*, *quality control and improvement*, or *public health research*. In addition, we provide a list of risk factors that determine the probability or magnitude of harm implied in *SeConts*. The risk factors provide a framework for assessing the privacy-related risks for patients implied in *SeConts*. We illustrate the use of risk assessment by applying it to a concrete example.

**Conclusions:**

In the future, research ethics committees and data use and access committees will be able to rely on and apply the framework offered here when reviewing projects of secondary use of clinical data for learning and research purposes.

## Introduction

The secondary use of clinical data for research purposes is increasingly recognized as a promising and crucial tool for improving health care and advancing medical research. Several initiatives strive to use data from medical care for secondary research and learning activities [1]. The US Institute of Medicine has called for a transformation toward a learning health care system (LHCS) to improve quality, expedite translation, and reduce costs [2]. The American Society of Clinical Oncology is pioneering the linkage of patient, provider, and research data with their quality improvement portal CancerLinQ [3]. In Germany, the national Medical Informatics Initiative aims to establish a national network of data integration centers to collect, aggregate, and analyze clinical data from all university hospitals in Germany.

There are numerous advantages of the secondary use of clinical data (ie, data derived from patient care) for research and learning activities. First, the clinical data are readily available. There is no need for any physical intervention or data collection through surveys. Costs for data aggregation, staff, and materials are considered to be low; data can be collected quickly [4]. Data have a high level of generalization due to the real-life setting in which they are collected, and this facilitates representative sampling while simultaneously increasing external validity [5,6]. Moreover, large sample sizes can be obtained by aggregating the data from different sites. For example, this benefits research on rare diseases [7]. Moreover, interventional studies that cannot be conducted prospectively due to ethical reasons may be performed retroactively [8], for instance, by systematically analyzing experimental therapies such as off-label use of drugs. Most importantly, patients can contribute their clinical data to research or learning activities without being exposed to immediate physical risks [5]. However, the secondary use of clinical data in research or learning activities entails data-associated risks that require further investigation.

This paper focuses on the most relevant aspects for patients in particular and for the trustworthiness and sustainability of secondary use of clinical data in general: risks concerning patients’ privacy and informational self-determination. The European Union General Data Protection Regulation (GDPR) requires data processors to carry out an “assessment of the impact of the envisaged processing operations on the protection of personal data” where there is a high risk to the “rights and freedoms of natural persons” (Article 35, 1, GDPR). An appropriate framework for risk assessment of the secondary use of clinical data in research or learning activities is lacking, as is a conceptual basis for such secondary data use. We aim to fill this gap by developing a framework for risk assessment that supports decision makers in research ethics committees and data use and access committees, as well as scientists, bioethicists, and funders who deal with the ethics and governance of secondary use of clinical data in data-gathering, non-interventional research or learning activities (ie, research and learning activities that rely solely on the collection of existing data). We will develop the risk assessment framework on a strong conceptual and empirical basis of two preceding elementary steps: (1) an analytical clarification of the concept of secondary use of clinical data in data-gathering, non-interventional research or learning activities (*SeConts*); and (2) an illustration of the types of research and learning activities that can be subsumed under this concept.

## Methods

In this study, we proceed in three main steps, each with a methodological approach. In *step one*, our methodological approach to clarify the concept of *SeConts* is to investigate the *intension.* In other words, what does the concept of *SeConts* mean? We first analyze each element of the concept (analysis) and then compile them for a comprehensive definition of the concept (synthesis). As the application of this methodological approach to clarify the concept cannot easily be separated from the result itself (the definitional clarification of the concept), we decided not to separate them and thus present both parts in the *Results* section.

Building on the clarification of the concept of *SeConts* (step 1), *step 2* examines its practical relevance. Regarding our methodological approach, we investigate the *extension* of the concept of *SeConts*, that is, the range of *objects* to which the concept can be applied. We examine concrete types of research or learning activities that can be subsumed under this concept. These types of research or learning activities were inferred from a scoping review (a), in which we searched PubMed and Google Scholar between October and November 2019 for bioethical literature that deals with the expected risks and benefits of secondary use of clinical data for biomedical research. The search terms used were *ethics*, *secondary use*, *re-use*, *clinical data*, *electronic health records*, *risks*, and *benefits* in different combinations. We limited our search to publications in English. We found numerous types of research or learning activities that, according to the authors, solely used clinical data. Next, we searched the biomedical literature for concrete studies (b) to find examples of previously identified types of research or learning activities. Relevant publications were identified after reading abstracts. After reading the full texts of the identified publications, we categorized them into the types of *SeConts* developed before (a).

On the basis of step 1 (conceptual clarification of *SeConts*) and step 2 (examination of the practical relevance of *SeConts*), we develop a framework for the systematic assessment of risks implied in *SeConts* in *step 3*. Operationalizing the general concept of risk to tailor it to *SeConts* presents a key methodological challenge when developing a risk assessment framework. We apply a widespread definition of risk as a *harmful event of a certain magnitude* that occurs with a certain *probability* [9-11]. According to this definition, risk assessment ideally results in a number. If we bet US $10 on a single number out of 37 in the roulette, the probability of losing is 1−(1/37) and the magnitude of harm is US $10. The resulting risk could therefore be quantified by the number of 9.7, which is 10×[1−(1/37)]. Although this quantitative understanding of risk is intuitively plausible, it is fraught with several difficulties in the context of *SeConts* concerning the quantification of *the probability* and *magnitude of harm*.

In terms of quantifying the *probability* of a harming event in the context of *SeConts*, there is neither a stochastic rule (as in the roulette example) nor empirical data that would allow an estimate of the probability of these events. Reports indicate that there have been data breaches (defined here as: *all types of events in which the confidentiality and planned protection of data is violated, whether through technical failure, human error, or deliberate unethical or criminal acts*) in the health care system in the past [12-14] that may suggest that such events could also occur in the context of *SeConts*. However, they do not allow for an assessment of their likelihood. Similarly, there is little information about the probability of further misuse of data stolen in the course of a breach. The *magnitude of harm* caused by the misuse of data is equally difficult to quantify. This is due to the fact that possible harm is predominantly of an immaterial social and psychological nature.

In the field of data science and in bioethics, various approaches have been developed to address the problem of operationalizing risks without recourse to quantification [15-20]. However, these approaches are neither designed nor transferable to *SeConts*. In this paper, we therefore propose a nonquantitative approach to operationalize both the *probability* and *magnitude of harm* for the assessment of risks within *SeConts*. We (1) identify risk factors in relation to data and their use within *SeConts* that increase or decrease the probability and magnitude of harm, (2) assess their individual severity, and (3) subsequently estimate the overall risk of a specific form of *SeConts*.

**Figure 1:**
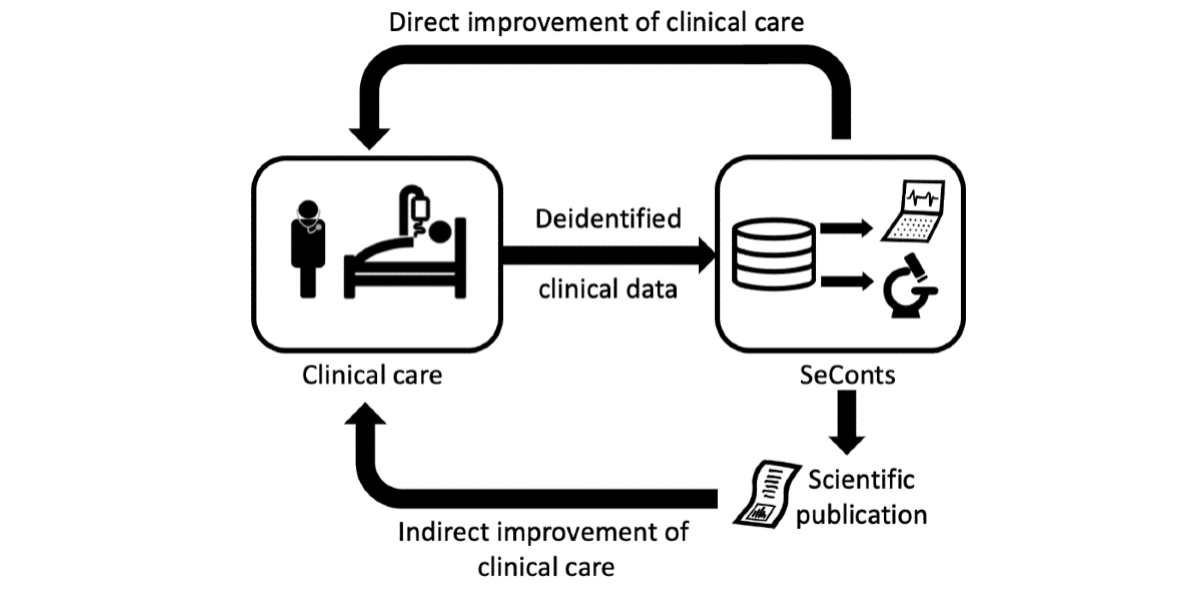
Flowchart of the secondary use of clinical data in data-gathering, non-interventional research or learning activities.

Similar to existing initiatives for secondary use of clinical data, such as the Medical Informatics Initiative [21] or CancerLinQ [3], we assume the following data flow from patients to *SeConts* (Figure 1): (1) Clinical data from electronic health records (EHRs) are deidentified, that is, identifying attributes (eg, names, addresses) are removed or replaced with a code. (2) Deidentified data are transferred to a central data warehouse and stored. (3) Deidentified data stored in the central data warehouse can be made available to researchers upon request to enable *SeConts*. Regarding risks to the confidentiality of EHRs that contain directly identifying data, there is evidence of leaks and attacks on EHRs. However, these risks affect every care unit that works with EHRs and are not specific to *SeConts*. Therefore, the risks to the confidentiality of EHRs are not the focus of our study.

Regarding the operationalization of *probability*, we assume that the most negative consequences for the individual patient are only expected if the patient can be identified from the data. Therefore, we assume unauthorized reidentification as a prerequisite for possible harm in connection with *SeConts*. According to our understanding, factors that increase the *probability of unauthorized reidentification* also increase the probability of misuse. We identify two categories of factors that directly or indirectly determine the *probability of unauthorized reidentification*: data-specific and contextual factors.

In terms of operationalizing the *magnitude* *of harm*, we distinguish different types of data according to their harm potential (see studies by Dyke at al [20] and Rumbold and Pierscionek [22]). To do this, we identify risk factors in the data that determine the extent of harm that may result from the misuse of the data. In other words, we provide an analytical answer to the question of what data aspects determine the degree of harm in the event of misuse. This approach is in line with the risk-based distinction between different categories of data in the GDPR (Article 9 GDPR).

How did we arrive at the factors that determine the *probability of unauthorized reidentification* and *magnitude of harm*? In the *first step*, we built on existing literature. As a basis for the factors determining the *probability of unauthorized reidentification*, we focused on the literature from different areas: literature on ethical, legal, and social implications of *SeConts*; literature on data security, data protection, and the assessment of reidentification risks; and national German and European data protection laws and regulations. For factors that determine the *magnitude of harm*, we analyzed the literature on genetic data that are generally classified as very sensitive, to learn what makes these data sensitive and transfer this knowledge, mutatis mutandis, on other types of data. In the *second step*, we extracted the individual factors—for both the *probability of unauthorized reidentification* and the *magnitude of harm*—from the literature and categorized them inductively. In the *third step*, we cross-referenced the factors with qualitative interviews conducted with experts from research, care, medical informatics, patient advocacy, and politics on the topic of perceived risk potentials of *SeConts*, part of which are to be published elsewhere [23]. In the presentation of the individual factors below, we refer to the literature on which they are based. Factors without a literature reference are taken from expert interviews that have not yet been published.

## Results

### Conceptual Clarifications and Definition of Secondary Use of Clinical Data in Data-Gathering, Non-Interventional Research or Learning Activities

Secondary use of health data is defined by the American Medical Informatics Association as “non-direct care use of PHI [personal health information] including but not limited to analysis, research, quality/safety measurement, public health, payment, provider certification or accreditation, and marketing and other business including strictly commercial activities” [24]. On the basis of a systematic scoping review, Robertson and colleagues categorized secondary use by distinguishing between four types of secondary use of clinical data: research, improving quality and safety of care, informing financial management, and education [25]. These and other similar classifications [26-28] help in understanding the broad spectrum of secondary uses of clinical data and illustrate that it is not limited to research. However, as pertains to the focus of this paper, that is, *the secondary use of clinical data in data-gathering, non-interventional research or learning activities*, the generic classifications lack further specifications and detail.

The term *secondary use* (or reuse) implies that there is also a primary use. Primary use encompasses the generation and use of data within the context of individual health care in hospitals and doctors’ offices to serve direct care needs. Secondary use refers to the use of these data for purposes other than individual care. Two points of criticism could be made against the distinction between primary and secondary use and the implied distinction between care and research. First, the distinction between care and research is notoriously difficult and widely discussed. Second, if secondary use of clinical data was to be implemented in the future as a standard in the health care system (ie, all health care data would be made available for possible research), this could lead to a tendency to collect data more systematically or collect more data than necessary in the care context, thereby blurring the distinction between data generation for care (primary use) and data generation for research (secondary use). In arguing against these two criticisms, however, we find that (1) ethical and legal codes are still fundamentally based on the distinction between care and research, which is unlikely to be abandoned in the coming years as blurred boundaries in governance and regulation are difficult to manage. Furthermore, we argue (2) that even if data are collected with the additional motivation of secondary use, the generation and collection of data would be driven by the primary goal of individual care.

*Clinical data* refers to data generated and collected in clinical contexts for patient care (diagnoses, anamnesis, treatment, medication, and so on) as well as data for accounting purposes or patient management, such as age, employment status, and other sociodemographic information. Clinical data also include data generated in the course of nonroutine treatment (off-label or experimental therapies) as long as it is collected for the primary objective of individual care. In terms of clinical data, we also include data reported from health care services and units to health insurance. In contrast to the widely used term health data, clinical data includes neither data gathered by (common, ie, nonclinical) smart devices and smartphone apps or research data understood as data generated for research purposes.

In contrast to research that generates data, *SeConts* only *gathers existing clinical data.* The gathering of data in *SeConts* can be done either by collecting clinical data of different patients from one institution or several institutions or by collecting only the results of analyses of clinical data carried out in the institution of primary use (decentralized analysis).

*SeConts* is *non-interventional* as it does not use data from interventions carried out with the aim or priority of data generation for research. *SeConts* solely uses existing data from medical care. The data used in *SeConts* may originate from interventions, but only from interventions carried out for the sake of individual diagnosis and treatment.

Both the terms *research* and *learning activities* refer to investigations in which the acquisition of generalizable knowledge beyond the needs and logic of individual care is the sole or primary intension. Research and learning activities cannot be clearly separated, but a rough distinction can be made in terms of their respective objectives. Research primarily aims at acquiring scientifically generalizable knowledge to be shared within the scientific community through scientific publications. Research usually indirectly improves health care and is realized through publications and by implementing practical conclusions based on research results. Learning activities, on the other hand, are designed to acquire knowledge about current care practices (eg, in a defined care unit) to derive appropriate and immediate measures to directly improve a specific health care unit or service. When talking about improving health care, the question arises whether *SeConts* has the potential to directly benefit the individual patient whose clinical data are reused. A direct (therapeutic) benefit is only possible under certain circumstances, for example, infection control of a clinical unit or research on a chronic disease. Younger patients with such a chronic disease could possibly benefit in several years from research that improves treatment of that very disease. However, as such benefits seem rather unlikely and exceptional, we understand *SeConts* as activities that are neither intended nor expected to directly benefit the individual patient whose data are used. In addition, there are ethical reasons for this narrow definition of *SeConts*, namely, to avoid any possibility of therapeutic misunderstanding.

In summary, the central concept of this paper, that is, *SeConts* can be defined as activities that:

1. exclusively use data produced for the purposes of and in the context of health care.
2. exclusively collect and do not generate data, that is, they are not based on data generated by interventions carried out primarily for the sake of research.
3. aim to acquire generalizable knowledge that goes beyond the needs and logic of individual care.
4. aim to directly improve health care units or services or publish their results for the promotion of biomedical science.

In a nutshell, *SeConts* describes *activities that solely use data produced for the sake of health care and in the context of health care to improve biomedical science or services.*

### Overview of Different Types of Secondary Use of Clinical Data in Data-Gathering, Non-Interventional Research or Learning Activities

Having clarified the concept of *SeConts* in the previous section, we now examine the scope and practical relevance of the concept of *SeConts* as previously defined. Which studies fall under the concept of *SeConts* (scope)? What is their practical relevance to medical research and improving medical care? We explore these questions in the next section. The literature review carried out to investigate the scope and practical relevance of research or learning activities that fall within our definition of *SeConts* (step 1) led to the following results: many types of research or learning activities common in the field of quality control and quality improvement can be subsumed under the concept of *SeConts* (in particular, under the term *learning activities*). Examples include *improvement of infection control*, which can be done by monitoring clinical data in hospitals to identify patients at high risk of infection [29]. Clinical data can also be used to create computerized algorithms for the *early detection of possible hazards from germs* [30]. These activities can be considered a component of the comprehensive ideal for transforming a particular health care institution into an LHCS.

At the national level, clinical data are used for *public health surveillance*. For instance, data from EHRs are searched for indicators of influenza in primary care to detect a pandemic in its early phase [31]. *Epidemiological studies* focus on the distribution of diseases as well as their causes and effects in populations, such as studies on the epidemiology of a certain infection to inform and improve vaccination initiatives [32]. In *outcomes research*, the effects (outcome) of certain interventions are investigated, such as the effects of a nationwide antismoking law on childbirth in the area of public health [33] or, on a clinical care level, evaluating the quality of care [34]. In *health services research*, investigators can use clinical data to explore the mean costs associated with (treatment of) a certain disease [35]. A well-established form of secondary use of clinical data is *registry studies* analyzing collections of data on all patients affected by a particular disease (registries) such as cancer registries [36].

Clinical data are also reused in clinical research in the form of *in-silico hypothesis testing*, where clinical trials are modeled with the help of data from EHRs [37]. Moreover, clinical data can be used for *comparative effectiveness research* [38] to compare “the benefits and harms of alternative methods to prevent, diagnose, treat, and monitor a clinical condition or to improve the delivery of care” [39]. *Evaluation of experimental therapies* can be conducted in terms of *SeConts*, for instance, by sharing data from single off-label (or compassionate) use from different hospitals. The secondary use of data from these therapies can help inform other physicians with similar patients about the course and outcome of different experimental therapeutic approaches [40]. Other studies that reuse clinical data in the sense of *SeConts* are *drug safety and efficacy studies* [41]. In addition, some basic research is conceivable using existing clinical data, such as *studies on risk factors* for certain diseases that can be linked to single influencing or moderating factors [42,43]. Another form of secondary use of clinical data lies in the area of *informatics research*, which uses clinical data to develop new software tools that have the potential to improve patient care or analyze and improve data security within a health system [44].

In addition to the different studies mentioned above, clinical data can be used in an *explorative* manner, which can be understood as encompassing three (potentially subsequent) steps. First, data can be analyzed (possibly using artificial intelligence) to *generate hypotheses* for future studies; second, in the case of new research ideas resulting from this, the data can be used to *check feasibility*; and third, to *identify potential participants* for recruitment of upcoming studies [45].

To further clarify the scope and concept of *SeConts* (by means of definitio ex negativo), we also want to mention two areas beyond the concept of *SeConts*: prospective clinical trials (eg, randomized controlled trials) and all other forms of studies that include interventions carried out for the sake of generating data; genome wide association studies in so far as they combine clinical data (phenotypes) with sequencing data generated for research purposes (genotypes).

Thus far, we have illustrated that there are many types of research or learning activities that can be conducted in the sense of *SeConts*. These types of research or learning activities display huge heterogeneity with respect to study designs, research questions, and contexts. Even the names of the types show this heterogeneity, as they refer to very different attributes describing the respective type of *SeConts*: some names refer to a specific method or study design (eg, in-silico hypothesis testing), whereas others refer to a subject area (eg, epidemiology). Given this heterogeneity, the question arises as to whether the different types of *SeConts* can be categorized in a way that allows for a better overview. The literature that attempts to capture the potential applications of secondary use of clinical data does not systematically distinguish between different types of research and learning activities [25,26]. Therefore, we searched for criteria or a particular logic that would provide some kind of categorization of the different types of *SeConts*. Some criteria that provide useful approaches to classification in other biomedical contexts are inappropriate. For example, although categorization by medical specialty seems conceivable at first glance, we determined it to be unhelpful on closer inspection. With all the different specialties that exist in medicine, such a categorization does not provide any reduction of heterogeneity. Categorization into common typologies of biomedicine, such as *basic research*, *clinical research*, and *translational research*, is also not useful, because learning activities, as described above, cannot be clearly assigned here. Ultimately, we found a categorization in terms of the *object of investigation* (ie, the entity about which *SeConts* seeks to produce knowledge) using three levels most appropriate. These three levels are familiar from the social sciences: micro level, meso level, and macro level. The object of investigation can be either patients (micro level), clinical care units such as clinical departments or single hospitals—a specific delimited patient population (meso level), or the general public (macro level). Taking the *object of investigation* as a criterion, we can attribute each type of research or learning activities to a general area of application: *non-interventional (observational) clinical research* focusing on individuals, *quality control and improvement* (and similar uses contributing to the creation of an LHCS) focusing on clinical units, and *public health research* focusing on the general public. Table 1 sums up all types of the aforementioned research or learning activities, including the object of investigation and area of application. It also shows that some types of research or learning activities are not limited to a single object of investigation or area of application.

**Table 1:** Possible types of research or learning activities within secondary use of clinical data in data-gathering, non-interventional research or learning activities.

Type of research or learning activities	Object of investigation	Area of application
Improvement of infection control	Clinical unit	Quality control and improvement
Early detection of possible hazards from germs	Clinical unit	Quality control and improvement
Public health surveillance	General public	Public health research
Epidemiology	General public	Public health research
Outcomes research	Patients or clinical unit	Public health research or quality control and improvement
Health services research	General public	Public health research
Register studies	General public	Public health research
In-silico hypothesis testing	Patients	Non-interventional (observational) clinical research
Comparative effectiveness research	Patients	Non-interventional (observational) clinical research
Experimental therapy evaluation	Patients	Non-interventional (observational) clinical research
Drug safety and efficacy studies	Patients	Non-interventional (observational) clinical research
Studies on risk factors	Patients	Non-interventional (observational) clinical research
Medical informatics research	Patients, clinical unit, or general public	Possible in all three areas of application
Explorative use	Patients, clinical unit, or general public	Possible in all three areas of application

### Tailored Framework for Risk Assessment of Secondary Use of Clinical Data in Data-Gathering, Non-Interventional Research or Learning Activities

#### Risks for Informational Self-determination and Confidentiality

In the previous section, we addressed two desiderata of the current literature on *SeConts* by defining our understanding of *SeConts* and clarifying its scope and practical relevance. These steps allow us to offer what we consider to be the third desideratum: a detailed analysis of risks for patients implied in *SeConts* as well as a framework to assess these risks. The risks to patients associated with the data are critical to *SeConts* because if they were to materialize, they could also have a major impact on the role, trustworthiness, and reputation of doctors, hospitals, and the public health system. *SeConts* implies potential risks for patients because of their (potentially reidentifiable and sensitive) clinical data being used. We assume that *SeConts* will usually rely on clinical data that cannot be classified as anonymous data. Even if direct identifiers are removed from data or replaced by a code (deidentification), the possibility of reidentifying a specific person in the data can rarely be excluded (see the list of potential risk factors below). In most cases, *SeConts* will use deidentified personal (or person-related) data; they imply risks for patients’ right to *informational self-determination*.

The term informational self-determination refers to “a person’s ability to freely decide whether and how personal data and information about her are collected, stored, multiplied, processed, and transferred by third parties” [46]. We regard informational self-determination as an instrumental value, that is, a means of protecting liberal individual and social rights and values such as equality or personal autonomy [47-49]. The protection of informational self-determination is particularly important in the health care sector: clinical data are highly sensitive as they contain information about the health status of a person and can therefore be highly susceptible to possible misuse. The confidentiality of the data is crucial for the patient-physician relationship and communication.

**Figure 2:**
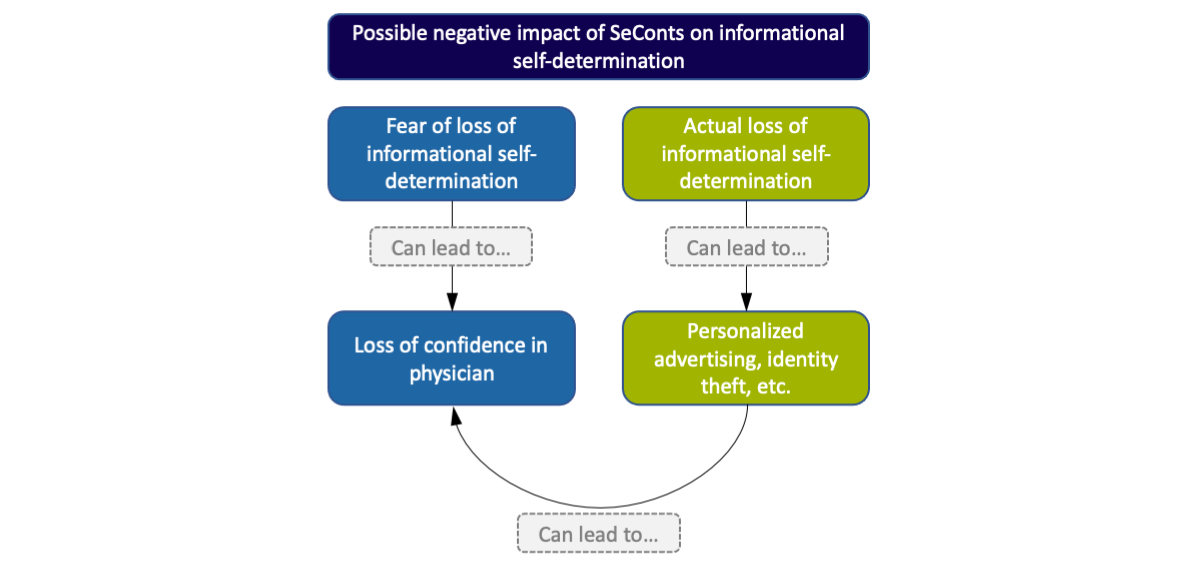
Possible negative impact of the secondary use of clinical data in data-gathering, non-interventional research or learning activities on the patient’s ability to exercise informational self-determination.

*SeConts* can have a negative impact on the patient’s ability to exercise informational self-determination in two ways (Figure 2): (1) the fear of a loss of informational self-determination and (2) its actual loss in case of data breaches in the meaning defined above (which, in turn, will most likely reinforce the fear of a loss of informational self-determination among patients).

With regard to fear (1), it is important to stress that informational self-determination is not only impaired if there are indications that third parties have accessed the data without authorization (and might or do use them against the data subject). Informational self-determination is already undermined if data subjects have reasons to develop a sense of vague uncertainty about the confidentiality of personal data [50] (chilling effect [51]). The fear of loss of informational self-determination alone can already have negative consequences. For instance, the fear that their data are not protected from unauthorized access can lead to patients not disclosing all the information that could be important for their personal care [52,53] due to a lack of confidence in their physician.

Concerning actual losses of informational self-determination (2), data breaches are a serious threat. A study carried out between 2010 and 2013 revealed a total of 949 data breaches in American hospitals involving almost 30 million patient records [14]. Even if the reported data breaches occurred in the context of patient care, and not in the context of *SeConts*, they uncover general problems with the protection of clinical data. As assumed by several authors [52,54,55], the negative consequences of data breaches, including unauthorized reidentification, could range from minor annoyances through personalized advertising to serious harms such as identity theft, stigmatization, blackmail, or discrimination, as well as other forms of data use without patient consent, such as the sale of data or use in studies in which patients have not consented.

#### Criteria to Assess Risk: Probability of Unauthorized Reidentification and Magnitude of Harm

**Figure 3:**
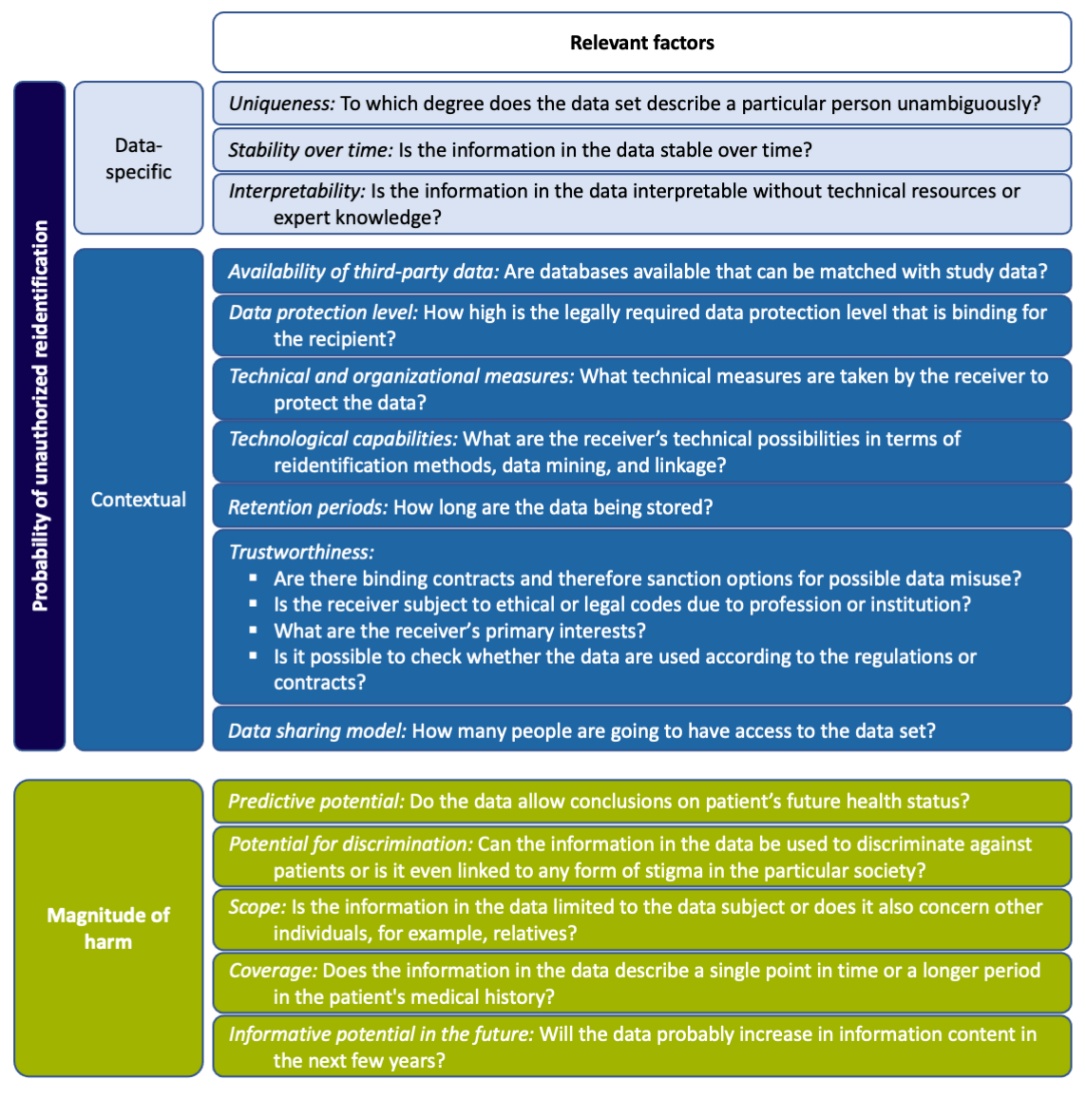
Factors of risk assessment for the secondary use of clinical data in data-gathering, non-interventional research or learning activities.

In the previous chapter, we outlined the potential risks to patients’ informational self-determination associated with *SeConts*. On the basis of our approach to assessing these risks (probability and magnitude of harm, see *Methods* section), we present a list of relevant factors that determine the *probability of unauthorized reidentification* and the *magnitude of harm*. These factors are partly interrelated and cannot always be clearly distinguished. Figure 3 provides an overview of the relevant factors, which we discuss in more detail below.

There are three data-specific factors that determine the probability of unauthorized reidentification:

1. *Uniqueness* of a data set [56,57]: Even after removing identifying attributes (name, date of birth, etc), unauthorized reidentification is possible, as the combination of attributes of a person’s medical record can be identifying. Therefore, the more unambiguously a person is represented in a data set by the combination of individual attributes, the higher the probability of unauthorized reidentification. Statistically, the smaller the number of cases in a data set, the more likely it is to correctly assign one data set to a specific individual. In addition, a larger number of variables (attributes of patients) in a data set allows for a more unique combination of attributes that again increases the probability of unauthorized reidentification [56,58,59]. Concepts such as *k*-anonymity [60], *l*-diversity [61], and*t*-closeness [62] aim to mitigate these risks by defining standards for data sets to reduce the probability of unauthorized reidentification.
2. *Stability over time* [63]: The more stable the attributes described in the data, the more likely it is to link the data set to a specific person. If an attribute described in the data is not permanent and is likely to change between each measurement (eg, blood pressure or blood glucose levels), it is impossible to use the associated data to uniquely assign it to an individual patient.
3. *Interpretability:* Certain types of data require special skills or technical tools to understand them. Thus, the degree to which data is easier or more difficult to interpret determines the number of people who are able to misuse the data. For example, anyone can interpret data relating to obesity, but laypersons are currently unable to interpret data from genetic sequencing or magnetic resonance imaging.
  However, the interpretability of data is a dynamic factor. Modern software development suggests that the interpretation of certain data might soon be supported by self-learning algorithms that may allow laypersons to interpret complex data such as genomic data or magnetic resonance imaging. *Interpretability* is directly related to the possible contexts in which data are used.

In addition to the data-specific factors regarding the probability of unauthorized reidentification in the context of *SeConts*, seven contextual factors must be considered.

1. *Third-party data:* Personal data of patients stored by third parties, such as voting registers or records from residents’ registration offices, can be used for reidentification when linked to clinical data [64-66]. Therefore, the availability of third-party data makes unauthorized reidentification easier [63], especially when the factor of uniqueness of the data set is high.
2. *Data protection level:* Every act of data exchange with other institutions or countries or jurisdictions brings with it the possibility of weaker data protection standards. The legally required data protection level that is binding to the recipient must therefore be taken into account [67].
3. *Technical and organizational measures:* Adequate measures need to be taken by the data recipient to protect the data. Here, the data protection level of the recipient, for example, according to approved codes of conduct (Article 40, EU-GDPR) or a data protection certification (Article 42, EU-GDPR), can be used as a benchmark.
4. *Technological capabilities:* The factor *technological capabilities* describes the technical means available to the respective data recipient to perform unauthorized reidentification. These include self-learning algorithms or other forms of artificial intelligence [68].
5. *Retention periods*: The longer the data are stored, the higher the risk of a data breach that can lead to unauthorized reidentification. Therefore, the retention (and deletion) periods specified by the data recipient are relevant factors in estimating the probability of unauthorized reidentification.
6. *Trustworthiness:* The factor *trustworthiness* is determined by several aspects [67]: the existence of binding (sanctionable) contracts that determine the terms of data use and access; the existence of ethical or legal codes on the part of the data recipient [22]; the primary interest of the receiver, for example, monetary or public interests; the verifiability of the use of the data in accordance with the applicable regulations or contracts.
7. *Data sharing model:* Depending on the data sharing model, data are disclosed to a different number of people; for example, data can be disclosed only to a small work group, a consortium, or can be uploaded to a public database. Every person with access to the data increases the probability of unauthorized reidentification.

Together with the aforementioned factors determining the probability of unauthorized reidentification, the following five factors must be considered to determine the magnitude of possible harm.

1. *Predictive potential:* The predictive potential of data refers to the extent to which a data set contains information that allows insights regarding future health status [69-71]. If a data set has a time-limited relevance, it can be considered less problematic than a data set that allows conclusions to be drawn about a person’s expected state of health for the next 30 years. Information with predictive potential could be, for example, the diagnosis of a chronic disease, but also the documentation of traumatic events that make a posttraumatic stress disorder diagnosis very likely within the next few years. In contrast, information about a fracture, for instance, does not necessarily provide information about future health status.
2. *Potential for discrimination:* Some data have obvious potential for discrimination because they contain information that can be used against individuals and groups. For example, it is conceivable that some employers discriminate against employees (eg, by not renewing contracts) if they know that the employee is affected by an illness (or has a disposition to illness) that is likely to lead to a longer absence of the employee in the future. Furthermore, stigmatization is possible [20]. For example, the disclosure of an HIV diagnosis can lead to stigmatization (through prejudice or social rejection).
3. *Scope:* The factor *scope* describes whether the information, and thus the possible risk in the data is limited to the data subject or whether it allows inferences about family members (eg, in the case of genomic information [69]) or colleagues (eg, information on diseases related to certain working conditions) [70].
4. *Coverage:* The factor *coverage* describes the data in terms of the period in which they were collected. Data from a single hospital visit cover a less extensive period than data documenting the entire medical history, for example, as collected by insurance companies. A complete medical history is likely to provide a more multifaceted picture of a person’s life than data collected on a single point in time and can therefore potentially cause greater harm in the event of misuse.
5. *Informative potential in the future:* Some data types can be expected to contain more information than can currently be made available. For example, we currently assume that the information content of genomic data is still largely unknown [70]. However, the constant progress in the field of genomic research suggests that in the future, we will be able to retrieve significantly more information from genomic data than is the case today, which can potentially cause greater harm in case of misuse.

## Discussion

### Application and Use of the Risk Assessment

After introducing the individual factors that determine the *probability of unauthorized reidentification* and the *magnitude of harm* implied by *SeConts*, questions arise concerning their application and use. How do these factors form a comprehensive framework for risk assessment in the context of *SeConts*? How can the framework be applied? How can the assessment results be used? Who may apply and use this framework?

With our risk assessment, we intend to support the analysis, evaluation, and potential decision-making process of research ethics committees and data use and access committees, as well as scientists, bioethicists, and funders investigating the ethical acceptability of requests for concrete types of *SeConts*. We recommend the following procedure with five consecutive steps to apply and use the risk assessment framework.

#### Application of the Risk Assessment (Steps 1 and 2)

The first two steps concern the application of the risk assessment framework:

- Step 1—*identification and evaluation of single risk factors:* A concrete projected study is examined in light of all factors determining the probability of unauthorized reidentification and magnitude of harm listed in Figure 3. The individual severity of each factor is evaluated, that is, whether and to what extent the factor is present and relevant in the specific study (plans) by classifying it as low, midrange, or high. We advise *against* converting these levels into numbers (eg, low=1, medium=2, and high=3), as this would falsely suggest a mathematical accuracy and cardinal order and could lead to a misinterpretation.
- Step 2—*comprehensive evaluation of risks for patients:* An overview of the evaluation of the single factors leads to a comprehensive picture of the risk profile of the concrete study. At this point, the methodological question of the relationship between the individual factors arises: Is the same importance attached to each factor, or are some factors considered more important than others and therefore given greater weight when moving from the estimates of the individual factors to a more comprehensive picture? We do not consider it plausible to state a priori that some factors are more important or should count more than other factors. As a default approach to a comprehensive evaluation of risks for patients in practice, we recommend that all factors be weighted equally. In particular circumstances, practical reasoning might suggest that the estimation of a single factor as *high* still does not adequately reflect the importance of that factor in the assessment of the comprehensive evaluation of risks for patients. In this case, more weight could be given to this factor, or it could even be treated as a decisive or exclusionary criterion. This might be appropriate, for instance, if a study plans to store very large and detailed sets of personal data in an open access data repository (factor *data sharing model*).

The fact that we advise against using numbers to evaluate the individual factors (step 1) already excludes the possibility of summing up numerical values as part of a comprehensive evaluation of risks for patients and presenting the risk (step 2) in a single number. Such a numerical approach would unreasonably suggest a mathematical or empirical reliability or precision that is not justified by the framework. Instead, a comprehensive evaluation of risks for patients is based on a rough summary of the evaluations of the single factors, considering their individual weight within the evaluated study. The results of the comprehensive evaluation of risks for patients can again be presented as low, midrange, or high, depending on the distribution of the individual factors.

#### Use of the Tailored Risk Assessment (Steps 3-5)

Steps 3-5 concern the use of the risk assessment framework:

- Step 3—*complete risk assessment:* It is important to be aware of the fact that the risk assessment addresses data-related risks for patients, which we deem to be the central and most important kind of risk from *SeConts*, but which are potentially still not the only kind of risk. Therefore, to gain a complete and comprehensive understanding, risks for other stakeholders (such as physicians and institutions) need to be taken into consideration.
- Step 4—*comprehensive ethical evaluation:* The complete risk assessment of a study is only one part of the comprehensive ethical evaluation. Typically, comprehensive ethical evaluation needs to include other aspects, in particular, the potential benefits of the envisaged study (to analyze and assess the risk-benefit ratio).
- Step 5—*reducing the risk profile by mitigating single factors:* The comprehensive ethical evaluation (step 4) can lead to three evaluation results of a planned study: (1) unethical and thus to be rejected, (2) ethically problematic but approvable under certain conditions, and (3) ethically sound and thus to be approved. In the case of (2), the applicant may be required to take specific measures to mitigate data-related risk to the data subjects. Tailored risk reduction measures should be chosen in light of the identification and evaluation of single risk factors (step 1) and against the backdrop of the comprehensive evaluation of risks for patients (step 2). Possible risk reduction measures may, for example, include modifying the data set to reduce the factor *uniqueness*, for example, by aggregating information (eg, age groups instead of age). In addition, special data sharing contracts can be applied to reduce the number of people who have access to the data (factor *data sharing model*).

#### A Practical Example of the Application of the Risk Assessment

**Figure 4:**
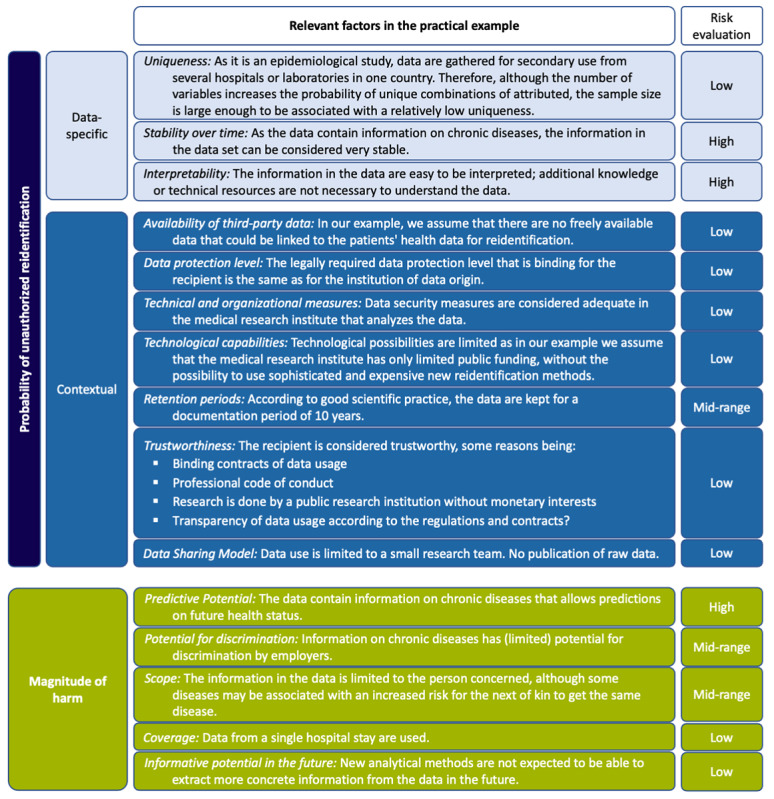
Practical example of a study on the epidemiology of Streptococcus pneumoniae infections.

After presenting the application and use, in the following section, we illustrate our risk assessment (steps 1 and 2) by applying it to a concrete study. Our example is a study on the epidemiology of Streptococcus pneumoniae infections [32] that we mentioned above in our list of examples for *SeConts*. According to the different areas of application (*non-interventional [observational] clinical research*, *quality control and improvement*, *public health research*) of *SeConts* we introduced above, the study can be classified as public health research. It gathers data from medical charts on “demographic characteristics, clinical syndromes, underlying conditions [eg, chronic diseases], and outcomes of illness” [32]. Figure 4 illustrates the application of the risk assessment framework for each factor (step 1) to a concrete example (For the sake of complete illustration of the risk assessment framework, we have added certain features to the study context where the study does not provide detailed information.).

In accordance with the application of our risk assessment approach, the results are as follows: (1) The probability of unauthorized reidentification can be considered relatively low because the majority of the relevant factors were assessed as low and (2) the magnitude of harm is in the midrange because most factors relevant to harm magnitude were assessed to be low or midrange. The comprehensive evaluation (step 2) shows that the overall risk for patients can be considered relatively low to midrange. After taking into account risks for other stakeholders (step 3), as well as the potential benefits of the study and other ethically relevant points (step 4), reviewers could request further mitigation of single risk factors (step 5). In our example, however, this is only possible to a limited extent because not all risk factors can be addressed without rendering the study itself impossible.

### Limitations

Having presented our risk assessment, its application and use, and illustrating its applicability by means of a concrete example, we will address the limitations and possible criticisms of our framework. Two objections can be raised against the risk assessment approach. First, we classify a priori certain data types according to their harm potential as a basis for operationalizing the *magnitude of harm*. However, it can be argued that such an a priori classification does not take into account the information that can be inferred from the data beyond the apparent information content [72,73]: Information that is considered potentially harmful (eg, sexual orientation, religious beliefs) can be derived from information that would probably be considered harmless a priori (eg, Facebook likes) [74]. We are aware that our a priori classification of data does not consider possible inferences that can be drawn from data in the future. However, there is an important difference in whether data contain information about a certain diagnosis or whether this diagnosis can only be assumed with a certain probability. Likewise, possible inferences that can be drawn from the data can rarely be predicted a priori. Therefore, our approach is limited to identifying the types of data that have a clear potential to cause harm themselves.

A second possible criticism of the proposed approach is conceivable. Regardless of whether information is inferred indirectly from data or whether the information is contained directly in it, the same data can entail different levels of individual risk for different people. Risk assessment, such as the one we present here, cannot reflect these different levels of individual risk. Both the probability of unauthorized reidentification and the possible magnitude of harm can be very different for the same data types in different people. The x-ray of a patellar luxation may be considered as nonsensitive information for most people, especially as it does not contain any identifying information. Nevertheless, a professional soccer player might disagree, as the information in the data contains the risk of unauthorized reidentification (due to possible analog and comparable x-rays of his club) and could be potentially harmful to his career (eg, through discrimination in relation to a possible contract extension). Our approach cannot depict these individually possible risks and can only provide guidelines for the assessment of generally expected risks. The risks of individual persons with special risk profiles must be addressed using individual measures. Among other things, this calls for the establishment of a suitable information and consent procedure or an opt-out option that allows persons with an individually high risk to decide for themselves whether this risk is too high or not. Which model of informed consent may be appropriate is not the subject of this study.

### Conclusions

In this paper, we addressed three desiderata of the current literature on *SeConts.* In the first step, we clarified the concept of *SeConts.* To this end, we analyzed each element of the concept and then provided a comprehensive definition of *SeConts* as *activities that solely use data produced for the sake of health care and in the context of health care to improve biomedical science or services.*

In the second step, we illustrated the scope and practical relevance of *SeConts* by providing a list of concrete types of research or learning activities that can be subsumed under the concept. These types of research or learning activities were roughly classified as either *non-interventional (observational) clinical research*, *quality control and improvement*, or *public health research*.

In the third step, we provided a framework for risk assessment for *SeConts*, focusing on the risks for patients related to informational self-determination. By operationalizing the concept of risk for application to *SeConts*, we identified factors that determine the *probability of unauthorized reidentification* as well as the *magnitude of harm* of a potential harming event implied in *SeConts*. We then discussed the application and use of our risk assessment framework and presented a practical example of a concrete study to illustrate its application.

Through our conceptual clarification of *SeConts*, we created a basis for understanding what *SeConts* means. The analysis of its scope shows that *SeConts* can realize its potential in a broad field of medical research. This illustrates the high practical relevance of *SeConts*. The risk assessment presented can be applied as an essential building block for an ethical evaluation of concrete *SeConts* conducted by research ethics committees and data use and access committees, as well as scientists, bioethicists, and funders. It can thus benefit the safe secondary use of clinical data in data-gathering, non-interventional research or learning activities.

## Abbreviations

DFG: Deutsche Forschungsgemeinschaft
EHR: electronic health record
GDPR: General Data Protection Regulation
LHCS: learning health care system
LinCDat: Learning from Clinical Data
SeConts: secondary use of clinical data in data-gathering, non-interventional research or learning activities
